# Assessing Meat Freshness via Nanotechnology Biosensors: Is the World Prepared for Lightning-Fast Pace Methods?

**DOI:** 10.3390/bios13020217

**Published:** 2023-02-02

**Authors:** Wen Xia Ling Felicia, Kobun Rovina, Nasir Md Nur ‘Aqilah, Joseph Merillyn Vonnie, Koh Wee Yin, Nurul Huda

**Affiliations:** 1 Faculty of Food Science and Nutrition, Universiti Malaysia Sabah, Kota Kinabalu 88400, Sabah, Malaysia; 2 Faculty of Sustainable Agriculture, Universiti Malaysia Sabah, Locked Bag No. 3, Sandakan 90509, Sabah, Malaysia

**Keywords:** hypoxanthine, xanthine, nanotechnology, meat freshness, biosensor

## Abstract

In the rapidly evolving field of food science, nanotechnology-based biosensors are one of the most intriguing techniques for tracking meat freshness. Purine derivatives, especially hypoxanthine and xanthine, are important signs of food going bad, especially in meat and meat products. This article compares the analytical performance parameters of traditional biosensor techniques and nanotechnology-based biosensor techniques that can be used to find purine derivatives in meat samples. In the introduction, we discussed the significance of purine metabolisms as analytes in the field of food science. Traditional methods of analysis and biosensors based on nanotechnology were also briefly explained. A comprehensive section of conventional and nanotechnology-based biosensing techniques is covered in detail, along with their analytical performance parameters (selectivity, sensitivity, linearity, and detection limit) in meat samples. Furthermore, the comparison of the methods above was thoroughly explained. In the last part, the pros and cons of the methods and the future of the nanotechnology-based biosensors that have been created are discussed.

## 1. Introduction

Over the years, the consumption of meat products has increased significantly across the globe [1]. Determination of meat quality is currently one of the most pressing concerns in the meat industry, along with maintaining the freshness and quality of the meat, as meat can be easily degraded during processing and storage [2]. It is challenging to evaluate the freshness of meat, as it involves changes in aging as well as meat spoilage-related microbiological, physicochemical, and biochemical characteristics [3]. Aging is a process that involves complicated changes in muscle metabolism in the post-slaughter process or storage phase, requiring the meat to achieve the optimal state for eating and determining meat tenderness [4]. ATP degradation is also one of the most important biochemical changes in the postmortem muscle of meat [5]. Adenosine triphosphate (ATP) is decomposed into adenosine-5′-diphosphate (ADP) and sequentially degrades to adenosine-5′-monophosphate (AMP), inosine-5′-monophosphate (IMP), inosine (HxR), hypoxanthine (Hx), xanthine (Xa), and uric acid (UA) [6,7]. The changes due to ATP degradation are considered an accurate way of evaluating the freshness of meat, as a high concentration of ATP catabolites correlates well with the loss of freshness in meat [8,9].

Numerous conventional techniques have been established for the detection of purine, including high-performance liquid chromatography (HPLC) [10,11], liquid chromatography-mass spectrometry (LC-MS) [12], UV visible spectrophotometry [13], and capillary electrophoresis (CE) [14]. Those techniques were used in the late twentieth century, and the majority of these techniques are still widely used today. For instance, HPLC is a highly sensitive and reproducible analytical technique [15]. It has the ability to quantify seven purine metabolites across the entire purine metabolic pathway and separate complex mixtures into their constituent parts [16]. Although the aforementioned techniques enable high selectivity and low detection limits, they can be extremely time-consuming, labor-intensive, resource-intensive, and require highly expensive equipment.

Given these drawbacks, there is a growing interest in using nanotechnology biosensors for the simple and real-time monitoring of the quality and safety of perishable food, with the goal of preventing food poisoning, food waste, and economic losses [17]. The emerging role of nanotechnology biosensors guarantees that everyone in the cold chain, from the consumer to the distributor, has easy access to data regarding the freshness of the food they are handling at any given time because of their simplicity, low cost, and satisfactory analytical performances. Recently, nanobiosensors have been implemented in the detection of purine derivatives, such as hypoxanthine, xanthine, and uric acid, to determine the freshness level of meat [8,18,19,20,21]. Nanobiosensors have not only been implemented in the meat industry, but have also been effectively applied in detecting endocrine-disrupting chemicals (EDCs) and heavy metals, such as Hg^2+^, in water [22,23]. These nanobiosensors showed excellent and satisfactory analytical performances in terms of their sensitivity, selectivity, linear range, and detection limit. Their validity for quantification of purine derivatives has been analyzed using conventional methods, and it shows that nanobiosensors are comparable to the purine derivatives detected by conventional methods [20,24,25].

**Figure 1:**
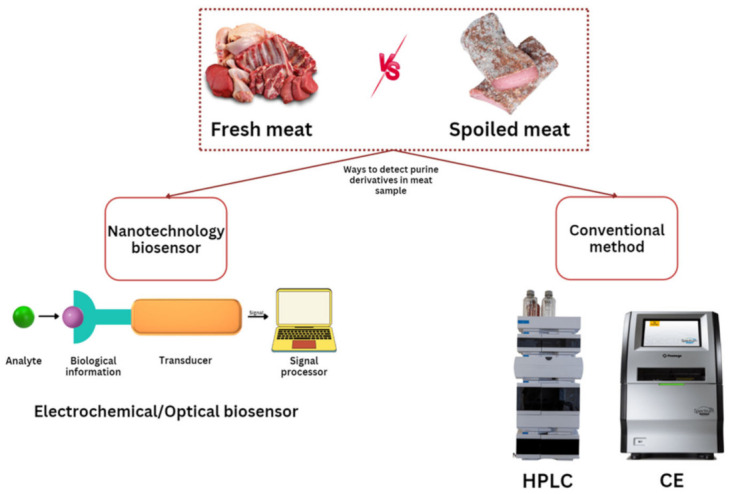
The available methods of biosensors for the detection of purine derivatives.

Herein, this article compares conventional techniques and nanotechnology biosensors for the detection of purine derivatives in determining the freshness of meat. Additionally, this study focuses on two of the most commonly reported approaches: electrochemical and optical methods utilizing different types of nanomaterials in meat freshness detection which summarize at Figure 1. The most recent works are presented, detailing not only the analytical performance parameters but also the application of the sensing platform to the analysis of real samples. Finally, concluding remarks and future perspectives are discussed.

## 2. Meat Freshness Indicators

Meat freshness can be defined in various ways. From the consumer’s point of view, the freshness of meat can be defined as an acceptable organoleptic property of tenderness, color, juiciness, and flavor [26]. Meanwhile, in the scientific aspect, the freshness of the meat is related to the microbial, chemical, sensory, and technological attributes of the meat [27]. Meat freshness can be evaluated using a microbial count; however, this method often requires a longer time to analyze the findings, as a bacterial count usually takes time for the incubation period [28]. The freshness of meat deteriorates due to the degradation of meat commonly caused by microbial, chemical, and pharmaceutical residue contamination [29]. Postmortem reactions, such as glycolysis, proteolysis, and lipolysis, also affect meat quality, usually occurring right after an animal is slaughtered [30,31].

After an animal is slaughtered, the skeletal muscle experiences physical and biochemical changes, thus, disrupting the stable interior environment and causing a depletion of blood flow and oxygen supply [32]. Physical and biochemical changes will continue to degrade or deplete even under chilled storage and, thus, affect the acceptability of the meat [33]. However, despite low blood flow and oxygen supply, the skeletal muscle does not stop producing ATP; synthesis of ATP occurs through the catabolism of stored glycogen, leading to lactate production and a lower pH of the meat [32]. Eventually, the ATP in the muscle will decrease and lead to the dysfunction of the sarcoplasmic reticulum, activating the ATP enzyme and decomposing the ATP into its metabolites [34]. The catabolic pathway of ATP generates metabolites, such as adenosine diphosphate (ADP), adenosine monophosphate (AMP), inosine monophosphate (IMP), inosine (INO), and hypoxanthine (Hx) [35]. Hypoxanthine (Hx) will further oxidize and, thus, produce xanthine (Xa) and uric acid (UA) [5]. The changes due to ATP degradation are considered an accurate way of evaluating the freshness of meat, as a high concentration of ATP catabolites correlates well with the loss of freshness in meat [36].

**Figure 2:**
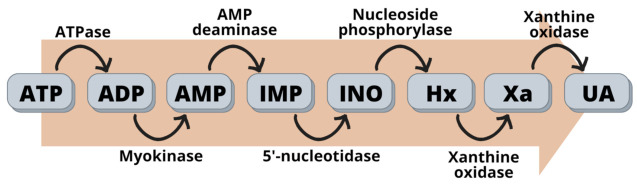
The catabolism of ATP with the respective enzymes responsible for the oxidation of each compound.

The concentration of IMP rose rapidly while ATP, ADP, and AMP were rapidly broken down [37]. The degradation of ATP to IMP is associated with the umami taste, which provides a pleasant savory taste to meat [21,38]. However, the disappearance of IMP and the formation of INO and hypoxanthine have always been connected to the loss of freshness in meat. Hypoxanthine commonly indicates the freshness of meat and the storage time of meat [39]. The catabolism of ATP forming IMP is mainly caused by enzymatic reactions; however, the hydrolysis of IMP forming INO and hypoxanthine is mainly affected by spoilage bacteria [6,40]. ATP-related compounds degrade rapidly in the presence of enzymes including ATPase, myokinase, AMP deaminase, 5′-nucleotidase, nucleoside phosphorylase, and xanthine oxidase [40,41,42]. The role of each enzyme in the catabolism of ATP to its metabolites is shown in Figure 2.

## 3. Conventional Techniques Used for Quantification of Purine Derivatives

To date, several traditional methods for quantifying purine derivatives in biological and food samples have been used, including high performance liquid chromatography (HPLC) [43,44,45], capillary electrophoresis (CE) [46], ultra-high performance liquid chromatography with mass spectrometry (UHPLC-MS) [47,48], gas chromatography-mass spectrometry (GC-MS) [49], mass spectrometry [50], and spectrophotometric methods [51], all of which provide high precision and sensitivity, and are summarized in Table 1. Despite their high selectivity and low detection limits, the above methods require highly expensive equipment and are extremely time-consuming, labor-intensive, human-intensive, and economically demanding [15,52].

### 3.1. High-Performance Liquid Chromatography (HPLC)

In recent times, liquid chromatography has increasingly become a standard technique. Liquid chromatography is usually coupled with mass spectrometry to provide a simple and robust interface. In both liquid chromatography and mass spectrometry, compounds are separated and identified based on the retention, efficiency, and selectivity of the separation mechanism [60]. Reversed-phase liquid chromatography or ion chromatography is usually used to analyze nitrogen bases, including purine and pyrimidine [61]. HPLC is often used to measure purines in food. It can be used on a wide variety of foods, including those that come from plants, fungi, and animals. Nevertheless, samples need to be pretreated appropriately. Furthermore, the chromatographic column, concentration of the mobile phase, pH, flow rate, column temperature, and wavelength of detection may influence quantification [62]. Therefore, the optimal conditions for chromatography are crucial in order to correctly quantify the particular compounds.

According to a recent study, the maximum number of purines in marine fish and aquatic products was estimated using HPLC with Agilent Eclipse XDB-C18 chromatography columns (4.6 mm × 250.0 mm × 5.0 mm) (Stevens Creek Blvd., Santa Clara, CA, USA) [63]. Adenine, guanine, hypoxanthine, and xanthine had LODs of 0.0774, 0.0178, 0.0118, and 0.0555 mg/L, respectively. Marine fish viscera contained significantly more purine than muscle, but had higher levels of adenine, guanine, and hypoxanthine. In another study, Qu et al. [45] used HPLC to detect four different purines in fish and shellfish and separated the purines into adenine, guanine, hypoxanthine, and xanthine. Excellent linearity was observed for adenine, guanine, hypoxanthine, and xanthine in the range of 0.05–300 mg/L, with the recoveries ranging from 91.5 to 105.0, respectively. Snails and shrimp contained more purines than fish and bivalves, and fish livers and skins contained more purines than muscles. Purines differ depending on the type of seafood. An analysis published by Fukuuchi et al. [53] determined 18 types of purines in three types of soup stocks (chicken consommé, dried bonito soup) by HPLC. There was the highest proportion of inosine monophosphate (IMP) in all umami soup stocks, followed by hypoxanthine, inosine, and guanosine monophosphate (GMP). Among the three soup stocks, dried bonito soup had the highest IMP content.

Hyperuricemia can be caused by consuming purine-rich foods. Previously, Larashinda et al. [54] analyzed the purine content of food from chicken in West Sumatra. Raw chicken meat contains a higher amount of purine than chicken rendang, chicken curry, chicken grilled, chicken balado, chicken seasoned, and chicken pop. Meanwhile, Vlassa et al. [16] simultaneously quantified allantoin, uric acid, xanthine, and hypoxanthine in cow milk by the HPLC-DAD method. In this experiment, an ODS-2 hypersil column was used as the chromatographic column and a 0.05 M (NH_4_)_2_HPO_4_ buffer solution (pH = 7.76) as the mobile phase. As indicated by the regression coefficient higher than 0.999, the HPLC-DAD validated method exhibited linearity, and the limits of detection and quantification ranged from 0.09 to 0.74 µg mL^–1^ and 0.27 to 2.24 µg mL^–1^. In addition, Wu et al. [55] developed a sensitive and accurate method of measuring purines and uric acid in Chinese chicken broth for hyperuricemia dietary management using high-performance liquid chromatography with variable wavelength detectors (HPLC-VWD). Chromatographic separation was performed on an Agilent TC-C18 (2) column (4.6 mm × 250 mm, 5.0 µm), using 0.02 M KH_2_PO_4_ (pH 4.0) as a mobile phase. In a recent study, Xiao et al. [56] used acidolysis and HPLC to compare the purine content of shiitake mushrooms after roast-drying, freeze-drying, and sun-drying. After roast-drying (120 °C), adenine levels decreased significantly, suggesting that DNA was damaged by thermal shock. Freeze-dried shiitake mushrooms had significantly less purine than roasted or sun-dried mushrooms.

### 3.2. Capillary Electrophoresis (CE)

Capillary electrophoresis (CE) is a method of separating compounds with different charges in a short period of time, particularly when they are known compounds. When CE is used to separate compounds, the peaks of the compounds can be compared with the peaks of the standards. Assays using CE are highly sensitive and repeatable, and they require little reagent, which reduces the cost [64]. Furthermore, CE is an automated machine that is handled through automated processes, making it a very useful machine in routine analytical work [46]. Separation of rich biological matrices by CE is equally common during proteolysis evaluations of dairy products [65] or meat [66]. Despite the foregoing, only a few studies on the use of CE for quality assessment of fish and meat products are available.

An electrophoresis method based on capillary zones (CZE) was developed by Zhu et al. [57] for the analysis of adenine nucleotides from yeast extracts. Based on its validation analyses, the method is relatively reliable and accurate, with high precision and recovery. Accordingly, Klampfl et al. [58] separated purine and pyrimidine bases in beer samples using the CZE technique and direct UV detection. Analytes were adequately separated from peaks associated with the sample matrix using the strongly alkaline carrier electrolyte based on diethylamine. Mu et al. [59] found four purines in soybean milk, including adenine, guanine, hypoxanthine, and xanthine, using the CE method with UV detection. The strongly alkaline carrier electrolyte based on DEA employed throughout this work provided sufficient separation of the investigated analytes from peaks related to the sample matrix. Various kinds of soybean milk samples were successfully analyzed using optimized acid hydrolysis followed by the CE method. A comparison was also made between the results obtained by the proposed CE method and those obtained by HPLC. Comparatively, CE performed well, and the quantitative results were similar to HPLC, while CE had the advantages of being significantly quicker and more affordable than HPLC.

## 4. Nanotechnology Biosensor Used in Determining Purine Derivatives

Nanotechnology involves the study of extremely small structures, between 0.1 and 100 nm in size, which have a variety of applications for sensor technologies [67]. The application of nanotechnology to biosensors forms the basis for developing nanobiosensors that are able to improve the sensitivity and specificity of biomolecule detection and show great potential in the food industry for diagnostics and biomolecular recognition [68,69]. Nanotechnology is gaining attraction in the food industry, as nanotechnology-based sensors are being used to measure food metabolites [70,71]. Biosensors are devices that use biological materials to detect and measure substances in a sample. These materials, called biorecognition elements, can include enzymes, antibodies, nucleic acids, cell receptors, micro-organisms, tissues, organelles, and natural products. They can also be biomimics, such as imprinted polymers, biomimetic catalysts, synthetic receptors, or combinatorial ligands. Alternatively, they can be biologically derived materials, such as functional nucleic acids, recombinant micro-organisms, or engineered proteins. The biorecognition element is combined with a physicochemical transducer, which is a device that converts the biochemical reaction into a measurable signal. This transducer can be electrochemical, optical, magnetic, thermometric, piezoelectric, or micromechanical in nature [72,73,74]. The selection of substrate to be fabricated onto the biosensor is important for inventing an efficient biosensor, as the dispersion of the sensing materials indicates the performance of the sensor [75,76]. According to previous studies, the employment of different nanomaterials (nanoparticles, carbon nanotubes (CNTs), quantum dots, and magnetic nanoparticles) led to the introduction of many new signal transduction technologies, as can be seen clearly in Table 2 and Table 3.

### 4.1. Electrochemical Biosensor

Electrochemical biosensors are becoming popular alternatives due to their good selectivity, sensitivity, and simplicity of operation. Not the least, the use of electrochemical biosensors can allow direct analysis of the analytes within a short time and is easy to conduct without trained personnel. Nowadays, food quality analysis or quality control departments require a fast response and field devices that can help detect certain contaminants present in the food. Foods, such as meat, are categorized as perishable foods, and the freshness of meat might degrade over storage time. Freshness indicators in meat can be measured by determining the concentration of hypoxanthine (Hx), xanthine (Xa), and uric acid (UA) in the meat [77]. Several methods have been developed to analyze and monitor the presence of purine derivatives in meat samples.

An electrochemical biosensor can be classified into three categories based on the type of signal it measures: amperometric, voltammetric, or conductometric. Amperometry measures the current flowing through a solution [78], voltammetry measures the potential of an electrode as a function of time [79], and conductometry measures the electrical conductivity of a solution [80]. In short, amperometry, voltammetry, and conductometry are all techniques used to measure the electrical properties of solutions and are often used in chemical analysis and in the development of sensors [81]. Additionally, enzyme sensors are often paired with amperometric and potentiometric electrochemicals because enzyme-based biosensors are highly sensitive and specific, portable, cost-effective, and can be miniaturized and tested at the point-of-care, which makes them increasingly attractive for clinical study, food safety management, or disease monitoring research purposes [82]. Table 2 summarizes the recent trends of electrochemical biosensors in detecting meat freshness and their analytical performance in food applications.

In recent years, various amperometric biosensors have been developed for the detection of xanthine and hypoxanthine as indicators of fish freshness. Amperometry is often used to measure the concentration of a specific ion in a solution. In amperometry, a potential is applied to an electrode, and the current that flows through the solution is measured [81]. The current is proportional to the concentration of the ion being measured. Amperometry is a sensitive technique that is particularly useful for measuring low concentrations of ions and detecting rapid changes in ion concentrations [78]. Recently, Wang et al. [83] designed an amperometry biosensor that has successfully immobilized xanthine oxidase on copper-based metal-organic (Cu-MOF) film to monitor the freshness of chilled squid and large yellow croaker. The designed XOD-electrochemical biosensor showed good sensitivity to hypoxanthine and xanthine, with a linear range of 0.01–10 μM. It also showed a low LOD of 0.0023 and 0.0064 μM for hypoxanthine and xanthine, respectively. Joon et al. [84] proposed an amperometric xanthine biosensor for fish meat freshness detection. It was developed by attaching xanthine oxidase nanoparticles (XODNPs) to gold (Au) electrodes. This biosensor showed a limit of detection and linearity of 0.01 μM and 0.01 to 1.0 μM, respectively. The use of xanthine oxidase nanoparticles in the construction of an amperometric xanthine biosensor eased its fabrication because XODNPs were immobilized directly onto polycrystalline Au electrodes via covalent coupling. Furthermore, hypoxanthine in fish samples can also be assessed via the designed biosensor composed of xanthine oxidase and uricase, which were immobilized on the surface of platinum via electrochemical polymerization of polypyrrole-paratoluenesulfonate (PPy-pTS). The developed amperometric biosensor showed a good LOD of 5 × 10^−6^ M and a wide linear working range from 5 × 10^−6^ to 5 × 10^−3^ M [85].

In addition, Sharma et al. [86] constructed an amperometric biosensor in which xanthine oxidase (XO) was extracted from *Bacillus pumilus* RL-2d and covalently immobilized onto screen-printed multiwalled carbon nanotubes with gold nanoparticle-based electrodes (Nano-Au/c-MWCNT). It revealed a good sensitivity of 2388.88 µA/cm^2^/nM and a LOD of 1.14 nM. Similarly, Albelda et al. [3] also fabricated an enzyme-based sensor of xanthine oxidase along with graphene/titanium dioxide nanocomposite (TiO_2_-G) and immobilized it onto the glassy carbon electrode. The sensor was used to determine the presence of hypoxanthine in meat. The fabricated Nafion/XOD/TiO_trip_-G/GCE sensor demonstrated a linear range of 20–512 μM with a LOD of 9.5 μM. Additionally, it was also revealed to have minimal interference from xanthine and showed a strong anti-interference towards uric acid, ascorbic acid, and glucose. These biosensors are therefore an effective and alternative way to assess the freshness of meat in an accurate manner. Tripathi et al. [19] also immobilized xanthine oxidase on the macroporous surface of a nickel oxide electrode prepared via a glancing-angle deposition. The developed biosensor showed a rapid response time of 7 s with a very high selectivity and sensitivity of 1.1 μA μM^–1^cm^–2^ towards xanthine. It also revealed good reproducibility with a superior LOD of 37 nM.

Complementarily, Sahyar et al. [87] immobilized xanthine oxidase onto a modified electrode of entrapped silver-doped zinc oxide nanoparticles (nano-Ag-ZnO) on a pencil graphite electrode electropolymerized by pyrrole. The biosensor showed excellent selectivity, with a superior sensitivity of 0.03 μA/mM and a LOD of 0.07 μM. Boluda et al. (2021) synthesized an amperometric biosensor via electrodeposition of ferrocenyl polycyclosiloxane polymers as a matrix for electrosynthesizing Pt nanoparticles. Xanthine oxidase was covalently immobilized onto the matrix and has achieved a good sensitivity towards xanthine with a linear range of 0.1–1.4 mM. Additionally, it also successfully applied the fish sample to detect xanthine as low as 48 nM. Those aforementioned biosensors have the same similarity, in that all the biosensors are immobilized with xanthine oxidase enzymes. The extensive usage of xanthine oxidase in assessing the freshness of meat is due to its biocompatibility, good mechanical performance, high activity, and stability, as well as its ability to produce a strong and reversible current in the presence of oxygen, which makes it suitable for use in biosensors [88]. Additionally, xanthine oxidase is an enzyme that catalyzes the oxidation of xanthine to uric acid and is important in the metabolism of purines in the body [89]. Therefore, xanthine oxidase is useful in the measurement of xanthine and uric acid concentrations.

However, there are also amperometric biosensors that do not immobilize enzymes on the matrix of the biosensor. Recently, Ahmad et al. [90] used sea-urchin-like cobalt-MOF (Co(TMA)MOF@CNF) on electrospun carbon nanofiber mat as a self-supporting electrode for sensing of xanthine (Xa) and uric acid (UA) in fish meat. Amperometric current response versus analyte concentration calibration plots were found to be linear over a board concentration range of 25–700 µM, with low detection limits of 96.2 nM and 103.5 nM for the Xa and UA, respectively. The sensor sensitivity for Xa is found to be 14.28 μA μM^−1^cm^−2^ and 5.78 μA μM^−1^cm^−2^ for UA. Co(TMA)MOF@CNF sensor provided acceptable results in detecting UA levels in the blood serum of gout patients, indicating its applicability for medical diagnosis. Meanwhile, Malhotra et al. [91] synthesized an amperometric biosensor for the detection of xanthine in chicken meat. The biosensors were composed of a 3-D self-supported electrocatalyst of cerium oxide nanocrystals doped with cobalt heteroatoms (CeO_2_-Co) and were homogeneously covered with 1-D tin oxide (SnO_2_) nanorods supported by a carbon cloth substrate. The proposed biosensor showed a wide linearity of 25–55 µM with a satisfactory LOD of 58 nM. Both electrospun carbon nanofiber mat and 1-D tin oxide (SnO_2_) nanorods have also been widely used in the development of amperometric biosensors due to their high surface area, which allows for the efficient immobilization of enzymes and other biomolecules, good electrical conductivity, which allows for the efficient transfer of electrons during the electrochemical reaction, and excellent stability, which makes them suitable for long-term use in biosensors. Despite their advantages, both CNF mat electrodes and SnO_2_ nanorods have some limitations in the development of amperometric biosensors. CNF mat electrodes are prone to aggregation, which can affect their performance in biosensors. SnO_2_ nanorods also have the tendency to aggregate and are sensitive to environmental conditions, which can also affect their performance in biosensors [92]. These revealed that biosensors that were constructed today have the potential to act as efficient sensors with great sensitivity and high accuracy in monitoring the food quality.

Another technique that is commonly used in electrochemical biosensors is voltammetry. Voltammetry is a versatile technique that can be used to measure the rate of a chemical reaction at an electrode and to determine the concentration of species in a solution. It is often used to study the electrochemistry of reactions and to develop sensors [93]. Recently, Khan et al. [94] detected xanthine in fish and meat samples using functionalized gold nanoparticles (GCE/PEDOT:PSS-AuNPs) assembled onto a host matrix of modified glassy carbon electrodes with poly(3,4-ethylenedioxythiophene) polystyrene sulfonate (PEDOT:PSS) nanocomposite. The proposed biosensor showed a linear range of 5.0 × 10^−8^ to 1.0 × 10^−5^ M and a good LOD of 3.0 × 10^−8^ M. Ghanbari and Nejabati [95] schemed a voltammetric sensor based on a reduced graphene oxide/chitosan/chromium oxide nanocomposite-modified glassy carbon electrode (GCE/rGO/CS/Cr_2_O_3_) to monitor the levels of dopamine (DA), uric acid (UA), xanthine (Xa), and hypoxanthine (Hx) in fish. The sensor revealed good sensitivity to DA, UA, Xa, and HX with a succession linear range of 5–160 μM, 10–500 μM, 10–400 μM, and 2–300 μM, along with LODs of 0.65 μM, 0.80 μM, 1.20 μM, and 0.85 μM, respectively. Similarly, Yazdanparast et al. [96] also immobilized xanthine oxidase on a glassy carbon electrode (GCE) composed of a nanobiocomposite of multiwalled carbon nanotubes (MWCNT) and poly (L-aspartic acid) (Poly (L-Asp)) film. The demonstration of the biosensor was successfully conducted by analyzing xanthine from the fish meat with a detection limit of as low as 3.5 × 10^−4^ μM and a linear range of 0.001–0.004 μM and 0.005–50.0 μM. Song et al. [25] constructed a novel biosensor based on ZnIn_2_S_4_/UiO-66-NH_2_ modified glassy carbon electrode (GCE) to detect xanthine and hypoxanthine in assessing freshness of fish. Incorporation of UiO-66-NH_2_ improved the functionality of the biosensor and provide a good simultaneous detection of xanthine and hypoxanthine with wide linear ranges of 0.025–40 µM and 0.3–40 µM along with a low LOD of 0.0083 µM and 0.1 µM.

The aforementioned techniques used glassy carbon electrodes (GCE) as the host matrix. Glassy carbon electrodes (GCEs) are widely used in biosensors due to their unique physical and chemical properties. GCEs have a large surface area, which allows for a large number of biomolecules to be immobilized on the electrode surface, leading to an increase in the sensitivity and selectivity of the biosensor [97]. In addition, GCEs have a low background current, making them ideal for electrochemical measurements as they reduce interference from other sources and provide more accurate measurements [98]. GCEs are also chemically stable, meaning they are resistant to degradation and can withstand harsh conditions, making them suitable for use in biosensors [99]. Furthermore, GCEs are biocompatible and do not interfere with biological processes, making them ideal for use in biosensors as they do not affect the activity of the biomolecules being measured [100]. Additionally, GCEs have a wide potential window, allowing for the measurement of a wide range of electroactive species, making them suitable for use in biosensors for the measurement of a wide range of biomolecules [97]. Overall, the unique properties of GCEs make them a valuable choice for use in biosensors.

There have been several efforts to design biosensors for the detection of hypoxanthine, xanthine, and uric acid in fish samples. Vishnu et al. [101] synthesized an enzyme-free biosensor composed of an Ag/AgCl electrode and a preanodized 4B grade pencil graphite electrode (PGE) for this purpose. The biosensor, referred to as Ag/AgCl (4B-PGE*), demonstrated linear calibration plots in the ranges of 6–30 μM, 8–36 μM, and 3–21 μM for hypoxanthine, xanthine, and uric acid, respectively, with detection limits of 1.09 μM, 0.40 μM, and 0.17 μM. Additionally, Li et al. [102] designed a simultaneous detector for xanthine and hypoxanthine to assess fish freshness. The sensor was based on Cu-BTC frameworks modified with carbon paste electrodes (CPEs) and prepared using triethylamine, copper (II) nitrate, and 1,3,5-benzenetricarboxylic acid (H_3_BTC). The sensor showed good linearity in the ranges of 5–8000 nM and 10–10,000 nM for xanthine and hypoxanthine, respectively, with limits of detection (LODs) of 0.89 nM and 2.1 nM. Pierini et al. [39] proposed a simple, rapid, and inexpensive method for detecting hypoxanthine, xanthine, and uric acid in fish samples using an edge-plane pyrolytic graphite electrode (EPPGE). The method had linear ranges of 0.1 to 50 μM for hypoxanthine and xanthine, and of 0.1 to 25.0 μM for uric acid, with LODs of 0.08, 0.06, and 0.03 μM, respectively. The low detection limit of the biosensor makes it suitable for use in quality control of meat samples by the food industry. Thakur et al. [18] immobilized a polyaniline-wrapped titanium dioxide (PANI@TiO_2_) nanohybrid as the sensing material onto an indium tin oxide (ITO) electrode to create a biosensor for xanthine. The biosensor had a detection limit of 0.1 µM and a linear range of 1 to 100 µM. It also showed a rapid response time, requiring only 10 s to detect the presence of xanthine.

**Table 2:** Recent trends of electrochemical biosensors in detecting meat freshness and their analytical performance in food applications.

Sensor	Detection Method	Nanomaterials	Samples	Analytes	Linear Range	LOD	Reference
Nafion/XOD/TiO_2_-G/GCE sensor	Voltammetry	Graphene/titanium dioxide nanocomposite (TiO_2_-G)	Pork tenderloins	Hx	20-512 μM	9.5 μM	[3]
XO-modified GLAD NiO electrodes	Conductometry	-	Fish	Xa	0.1–5 μM	37 nM	[19]
XOs/PANI@ TiO_2_/ITO electrode	Differential pulse voltammetry	Polyaniline-wrapped titanium dioxide (PANI@TiO_2_) nanohybrid	Rohu (*Labeo rohita*) fish	Xa	1–100 µM	0.1 µM	[18]
Au-PEDOT- fMWCNT/ GCE	Conductometry	Functionalized MWCNT-nanogold	Fish meat	UA Xa Hx	0.1–800 μM 0.05–175 μM 0.1–150 μM	199.3 nM 24.1 nM 90.5 nM	[20]
ZnIn_2_S_4_/UiO-66-NH_2_/GCE	Amperometry	-	Large yellow croaker	Hx Xa	0.3–40 µM 0.025–40 µM	0.1 µM 0.0083 µM	[25]
EPPGE	Amperometry	-	Fish	Hx Xa UA	0.1–50 μM 0.1–50 μM 0.1–25.0 μM	0.08 μM 0.06 μM 0.03 μM	[39]
XOD-Cu-MOF	Amperometry	Metal organic framework nanofibers (MOF)	Chilled squid Large yellow croaker	Hx Xa	0.01–10 μM	0.0023 μM 0.0064 μM	[83]
XODNPs/Au	Differential pulse voltammetry (DPV)	Nanoparticles of xanthine oxidase (XODNPs)	Fish meat	Xa	0.01–1.0 μM	0.01 μM	[84]
Pt/PPy-pTS-XnOx/U	Cyclic voltammetry	-	Fish meat	Hx	5 × 10^−6^–5 × 10^−3^ M	5 × 10^−6^ M	[85]
XO/nano-Au/c-MWCNT	Amperometry	Screen-printed multi–walled carbon nanotubes (c-MWCNT) gold particle	Fish and chicken meat	Xa	2388.88 µA/cm^2^/nM	1.14 nM	[86]
XO/nano Ag–ZnO/PPy/PGE	Cyclic voltammetry	Silver-doped zinc oxide nanoparticle (nano Ag-ZnO)	Sea bass fish	Xa	0.06–0.6 μM	0.07 μM	[87]
MFPP/FPP/PtNPs	Amperometry	Platinum nanoparticles (PtNPs)	Fish	Xa	0.1–1.4 mM	48 nM	[88]
Co(TMA)MOF@CNF	Amperometry	Carbon nanofibers (CNFs) Cobalt-metal organic framework (MOF)	Fish meat	Xa UA	25–700 µM	96.2 nM, 103.5 nM	[90]
SnO_2_@CeO_2_-Co	Differential pulse voltammetry	Cerium oxide nanocrystals doped with cobalt heteroatoms (CeO_2_-Co) and Tin oxide (SnO_2_) nanorods	Chicken	Xa	25 nM–55 µM	58 nM	[91]
GCE/PEDOT:PSS-AuNPs	Voltammetry	Functionalized gold nanoparticle	Fish and meat	Xa	5.0 × 10^−8^–1.0 × 10^−5^ M	3.0 × 10^−8^ M	[94]
GCE/rGO/CS/Cr_2_O_3_	Amperometry	Reduced graphene oxide	Fish meat	DA UA Xa Hx	5–160 μM 10–500 μM 10–400 μM 2–300 μM	0.65 μM 0.80 μM 1.20 μM 0.85 μM	[95]
XO/Poly(l-Asp)/MWCNT/GCE electrode	- Cyclic voltammetry (CV) - Differential pulse voltammetry (DPV)	Multi-walled carbon nanotube (MWCNT)	Fish meat	Xa	0.001–0.004 μM	3.5 × 10^−4^ μM	[96]
Ag/AgCl (4B-PGE*)	Amperometry	-	Freshly dead fish	Hx Xa UA	6–30 μM 8–36 μM 3–21 μM	1.09 μM 0.40 μM 0.17 μM	[101]
Cu-BTC/CPE	Amperometry	-	Fish	Hx	5–8000 nM	2.0 nM	[102]

In addition to the widely used glassy carbon electrodes, other electrode materials have also been widely employed as the host matrix in electrochemical biosensors, including pencil graphite electrodes, carbon paste electrodes, edge-plane pyrolytic graphite electrodes, and indium tin oxide. Pencil graphite electrodes, created from finely ground graphite, are commonly used as a reference electrode in electrochemical measurements [103]. Carbon paste electrodes, on the other hand, are created by mixing carbon powder with a binder, such as graphite or petroleum jelly, and forming the mixture into a paste [104]. These electrodes are often used in electrochemical sensors and biosensors because they have a large surface area and are easy to fabricate [105]. Edge-plane pyrolytic graphite electrodes, also known as EPPGE, are created from high-quality, highly ordered pyrolytic graphite and have a highly smooth and flat surface [106]. This makes them useful for electrochemical measurements that require a stable and low-noise reference electrode [107]. Meanwhile, indium tin oxide (ITO) electrodes are created from a transparent, conductive oxide of indium and tin and are commonly used in electrochemical sensors and biosensors because of their high conductivity and transparency. In terms of performance, pencil graphite electrodes have a relatively high resistance and may not be suitable for use in high-frequency or high-current applications [103]. Carbon paste electrodes have a higher conductivity than pencil graphite electrodes, but their performance can be affected by the binder used and the particle size of the carbon powder [104]. EPGE electrodes, on the other hand, have very low resistance and are highly stable, making them suitable for use in a wide range of electrochemical measurements, including high-frequency and high-current applications [106]. ITO electrodes have high conductivity and transparency, making them useful for applications where both electrical conductivity and optical transparency are required, such as electrochemical sensors and biosensors for in vitro and in vivo measurements [108,109]. Overall, the choice of electrode material will depend on the specific requirements of the electrochemical measurements being performed [108]. It is important to consider factors such as conductivity, stability, and compatibility with the measurement system when selecting an electrode [105].

Nowadays, researchers frequently employ nanomaterials on the host matrices of biosensors to improve their sensitivity and selectivity. Several nanoparticles have been employed in the development of the electrochemical biosensor, as can be seen in Table 2. For instance, Wang et al. [110] proposed a dual enzyme electrochemiluminescence sensor to assess the presence of inosine 5′-monophosphate (IMP) in meat. Silver nanoparticles (AgNPs) were obtained via reduction of luminol and were mixed with zeolite imidazole backbone-67 (ZIF-67) to successfully produce ZIF-67@AgNPs-Luminol powder. The prepared sensor showed a linear range of 0.003–25 g/L with a satisfactory LOD of 0.0017 g/L. Silver nanoparticles have high conductivity and surface-enhanced Raman scattering (SERS) activity, making them suitable for use in electrochemical sensing applications [111]. There are several potential advantages to using silver nanoparticles on MOFs for electrochemical biosensing. Firstly, the high conductivity of silver nanoparticles can improve the sensitivity of the biosensor, as it allows for faster electron transfer between the electrode and the analyte [112]. In addition, the SERS activity of silver nanoparticles can enhance the signal of the biosensor, allowing for the detection of trace amounts of analytes [113]. Secondly, the tunable pore size of MOFs can be used to selectively capture specific analytes based on their size [114], while the functionalization of the MOFs with biomolecules can allow for the selective detection of specific analytes [115]. The combination of these two factors can improve the selectivity of the biosensor. Finally, the high stability of MOFs under a wide range of conditions, combined with the high stability of silver nanoparticles [113], can result in a biosensor with stable performance over a long period of time [111]. However, it is important to consider factors such as the synthesis and stability of the silver nanoparticles as well as the compatibility of the MOFs with the silver nanoparticles [114] in order to optimize the performance of the biosensor.

Conductometry is a type of electrochemical biosensor that is often used to determine the concentration of ions in a solution and to study the properties of electrolytes. It is particularly useful for measurements where the concentration of ions is expected to vary widely [116]. In addition to amperometry and voltammetry techniques, conductometry is a simple and rapid technique that is well-suited for a range of applications. To illustrate the potential of conductometry, Sen and Sarkar [20] employed the technique to electrodeposit a nanogold-decorated PEDOT:TOS polymeric nanocomposite onto functionalized multiwalled carbon nanotubes (fMWCNTs). This process increased the conductivity of the synthesized film, which was then used to detect hypoxanthine, xanthine, and uric acid in human serum, urine, and fish samples. The film exhibited good sensitivity, with values of 1.73, 14.31, and 3.82 μA μM^−1^cm^−2^, as well as linear ranges of detection of 0.1–800, 0.05–175, and 0.1–150 μM, respectively. Additionally, the film had limits of detection (LODs) of 199.3, 24.1, and 90.5 nM for uric acid, xanthine, and hypoxanthine, respectively. It is worth noting that the incorporation of nanomaterials into host matrices can enhance the sensitivity and selectivity of biosensors. In summary, each of these techniques has its own advantages and limitations, and the most appropriate technique will depend on the specific requirements of the measurement. Amperometry is sensitive and useful for measuring low concentrations of ions, while voltammetry is versatile and useful for studying electrochemical reactions and developing sensors. On the other hand, conductometry is simple and rapid, and it is particularly well-suited for measuring ion concentrations over a wide range.

### 4.2. Optical Sensor

Optical sensors have attracted a great deal of attention among researchers for analytical applications, such as monitoring and evaluating the biological as well as the chemical substances. Optical sensor array technology offers visual adjustments that mimic human smell to detect, classify, and interpret complex gases and volatility based on chem-responsive colors. The optical sensor array is more objective than human olfaction and electronic noses and is not prone to interference, as human olfaction leads to subjective results depending on one’s preferences [117]. Additionally, optical sensor arrays have been increasingly implemented in food science and industry for their visualization, rapidity, and nondestruction in research. Optical sensors are related to the interaction of biorecognition elements, such as enzymes, antibodies, antigens, whole cells, or nucleic acids, with the optical field. Generally, optical biosensing is categorized into two general modes: (1) label-free: it detects a signal directly, and (2) label: it requires the use of a label and follows an optical signal generated by a colorimetric, fluorescent, or luminescent method, respectively [118]. Optical biosensors provide advantages over conventional techniques in offering real-time results, rapid analysis completion, high sensitivity and specificity, low concentration and amount of analyte required, a reusable sensor chip, and all of this in a cost-effective manner. Optical biosensors are widely implemented in various fields, including healthcare, environmental analyses, biotechnology industries, and the food industry.

Optical biosensors’ working principles are the same as those of electrochemical biosensors. An optical biosensor is a compact analytical device integrated with an optical transducer system that contains biorecognition sensing features. The fundamental objective of an optical biosensor is to generate a signal that is directly proportional to the amount and concentration of a measured analyte. The biomolecules used on the functionalized surface of optical biosensors can either be: (1) enzymes, (2) antibodies, (3) antigens, (4) whole cells and tissues, (5) nucleic acids, or (6) receptors. Biorecognition molecules are put in close proximity with the optical transducer, such as surface plasmon resonance (SPR), a refractometer, resonators, gratings, or interferometers, to detect the chemical interaction of biorecognition molecules with the targeted analytes [119,120]. Fluorescence analysis has become the most effective bioanalytical device as compared to other types of biosensors due to its inherent benefits, high sensitivity, and efficiency of anti-interference [121]. Table 3 summarizes the recent trends of optical biosensors in detecting meat freshness and their analytical performance in food applications.

Fluorescence optical sensors are a type of biosensor that uses the fluorescence of a substance to detect its presence and concentration. Fluorescence occurs when a substance absorbs light of a certain wavelength and then re-emits it at a longer wavelength. Fluorescence sensors measure the intensity of the emitted light, which is proportional to the concentration of the substance in the sample. Fluorescence sensors are widely used in various applications, including environmental monitoring, food and beverage testing, and medical diagnostics. They are sensitive to a wide range of wavelengths and can be used to detect substances that are not visible to the naked eye [122]. Hu et al. [123] developed a sensing platform based on aminofunctionalized metal-organic framework nanosheets with peroxidase mimic enzymes and fluorescence properties for the detection of hypoxanthine in fish samples. The NH_2_-Cu-MOF nanosheet showed a linear relationship with the concentration of hypoxanthine in the range of 10–2000 μM with 3.93 μM of the limit of detection. This biosensor was successfully applied to detect hypoxanthine in the fish samples with satisfactory results and has a promising prospect for target detection in food analysis. Additionally, Liu et al. [124] used a novel, simple, sensitive, and reliable fluorescence sensor based on S1 nuclease, FAM-labeled ssDNA (DNA-F), and graphene oxide (GO) to detect beef meat freshness in the presence of adenosine triphosphate (ATP). Under the optimal conditions, a linear correlation between the fluorescence and the ATP concentration from 20 μM to 3500 μM was obtained with a detection limit of 3.2 μM. Chen et al. [121] developed a fluorescence biosensor using platinum nanoparticles (Pt NPs), containing a peroxidase mimicking activity for rapid detection of hypoxanthine in aquatic products including fish, shrimp, and squid samples. A linear relationship was shown between the fluorescence intensity and the hypoxanthine concentration in the samples with values ranging between 8 μM and 2500 μM. The detection limit of the Pt NPs fluorescence biosensor was as low as 2.88 μM with excellent recovery rates of 103.94–109.00%.

Zhang et al. [10] developed a fluorometric assay using an AIE-active probe, TPE-HPro, and the fabrication of xanthine oxidase to detect hypoxanthine in artificial urine samples. The linear range of the quantification of hypoxanthine was up to 120 μM with a limit of detection of 1.2 μM, and it was proven to be matched with the endogenous hypoxanthine levels in human plasma and urine. The limit of detection of 1.2 μM is relatively low, indicating a high sensitivity of the biosensor. The linear range of up to 120 μM is relatively wide, indicating a good accuracy and precision of the biosensor in measuring the concentration of hypoxanthine over a wide range. Mou et al. [24] designed an orange emissive carbon dots (O-CDs) sensor with a high fluorescence quantum yield for the detection of hypoxanthine in fish. The fabricated O-CDs sensor demonstrated a linear range of 2–250 μM with a detection limit of 0.61 μM. The limit of detection of 0.61 μM is lower than that of the biosensor developed by Zhang et al. [10], indicating a higher sensitivity to the O-CDs sensor. The linear range of 2–250 μM is slightly narrower than that of the biosensor developed by Zhang et al. [10], indicating slightly lower accuracy and precision of the O-CDs sensor in measuring the concentration of hypoxanthine. Zhao et al. [125] determined hypoxanthine in fish using cysteine-functionalized copper nanoclusters (Cys-CuNCs). This fluorescence method showed a limit of detection and linearity of 0.7 μmol/L and 8–400 μmol/L, respectively. The limit of detection of 0.7 μmol/L is lower than those of the biosensors developed by Zhang et al. [10] and Mou et al. [24], indicating a higher sensitivity of the Cys-CuNCs sensor. The linear range of 8–400 μmol/L is slightly narrower than that of the biosensor developed by Zhang et al. [10] but wider than that of the biosensor developed by Mou et al. [24], indicating good accuracy and precision of the Cys-CuNCs sensor in measuring the concentration of hypoxanthine.

Xue et al. [126] constructed a novel, simple, and efficient xanthine biosensor to indicate the level of freshness of fish. The biosensor assay was composed of zinc oxide nanomaterials and xanthine oxidase, which were reported to provide specific and quantitative xanthine detection. The XOD@ZnO biosensor has been shown to have a wide linear range of 2.67 × 10z^−6^ to 2.67 × 10^−4^ mol L^−1^ with a sufficiently low detection limit of 1.30 × 10^−10^ mol L^−1^. The low detection limit and wide linear range of the XOD@ZnO biosensor indicate high sensitivity, accuracy, and precision in detecting and measuring the concentration of xanthine in fish samples. Luo et al. [127] designed a portable fluorescence/colorimeter hydrogel based on silver metallization to monitor the biogenic amines (BAs) in fish. The portable functional hydrogel was fabricated by using only silver ions and β-D-GP in the agarose gel. The detection limit of 2.77 × 10^−9^ mol dm^−3^ was calculated with a linear range of 28.5 × 10^−9^ to 114.9 × 10^−9^ mol dm^−3^. This method provides a simple, low-cost, and sensitive method for nondestructive, real-time monitoring of the fish’s freshness. The low detection limit and wide linear range of the portable functional hydrogel indicate high sensitivity, accuracy, and precision in detecting and measuring the concentration of BAs in fish samples. In terms of analytical performance, both the XOD@ZnO biosensor and the portable functional hydrogel have low detection limits and wide linear ranges, indicating high sensitivity, accuracy, and precision in detecting and measuring the concentrations of xanthine and BAs in fish samples, respectively. However, it is worth noting that the XOD@ZnO biosensor is specifically designed for the detection of xanthine, whereas the portable functional hydrogel is designed for the detection of BAs, which are a group of nitrogenous compounds that are produced during the decomposition of proteins in fish and are used as indicators of fish freshness. Therefore, the specific analytical performance of the XOD@ZnO biosensor and the portable functional hydrogel should be compared within the context of their respective applications.

A colorimeter is a type of optical sensor that uses colorimetry, a method of measuring the concentration of a substance by measuring the intensity of its color, for detecting and quantify the presence of a substance in a sample. They are simple, inexpensive, and easy to use, and they can detect a wide range of substances, including chemicals, biomolecules, and pathogens [128]. Hsu et al. [129] proposed silver nanoplates (AgP) as the colorimetric sensing platform to detect xanthine in fish meat. Detection of xanthine was conducted via the etching process of AgP particles/aggregation/fusion steps, which caused a change in color from blue to gray. The linear response range of xanthine was from 0.15 μM to 0.60 μM, with a satisfactory limit of detection (LOD) of 0.011 μM. The low LOD and narrow linear range of the AgP colorimeter indicate high sensitivity and good accuracy and precision in detecting and measuring the concentration of xanthine in fish meat. Chen et al. (2017) developed a simple, visual, and economical biosensor fabricated with gold nanorods (GNRs) to evaluate the freshness of fish with the naked eye. Hypoxanthine in fish samples is dissolved in oxygen, thus, reacting with xanthine oxidase to produce H_2_O_2_ in the presence of Fe^2+^. The linear range of hypoxanthine in GNRS/SPR is from 0.05 mM to 0.63 mM with a correlation coefficient of 0.9945. However, it has been shown that hypoxanthine is not only found in meat but also in urine samples. The narrow linear range and high correlation coefficient of the GNRS/SPR colorimeter indicate good accuracy and precision in detecting and measuring the concentration of hypoxanthine in fish samples. Mooltongchun and Teepoo [130] developed a fast, sensitive, and cost-effective paper-based colorimetric biosensor (lab-on-paper) for enzyme catalytic reactions (xanthine oxidase) to detect hypoxanthine in fresh and processed meat samples within 5 min. The technique has been validated against spectrophotometric detection and has shown good accuracy, as well as the fact that no specialized tools are needed, providing an alternative to traditional techniques. The linear range of hypoxanthine is 5–40 mg/L with a detection limit of 1.8 mg/L. The low detection limit and wide linear range of the lab-on-paper colorimeter indicate high sensitivity and good accuracy and precision in detecting and measuring the concentration of hypoxanthine in fresh and processed meat samples. In terms of analytical performance, all three colorimeters described previously have good sensitivity, accuracy, and precision in detecting and measuring the concentration of xanthine or hypoxanthine in different types of samples. The AgP colorimeter has a low LOD and narrow linear range, indicating high sensitivity and good accuracy and precision in detecting xanthine in fish meat. The GNRS/SPR colorimeter has a narrow linear range and a high correlation coefficient, indicating good accuracy and precision in detecting hypoxanthine in fish samples. The lab-on-paper colorimeter has a low detection limit and a wide linear range, indicating high sensitivity and good accuracy and precision in detecting hypoxanthine in fresh and processed meat samples.

Wang et al. [131] used the peroxidase-like activity of cobalt-doped graphite phase carbon nitride (Co-doped-g-C_3_N_4_) as a colorimetric method in determining the presence of hypoxanthine (Hx) in aquatic products, such as fish. Based on the research, Hx can be directly detected using the spectral absorbance at 652 nm with a limit of detection (LOD) of 1.84 mg/kg and a linear range of 2.50–153.1 mg/kg. The low LOD and wide linear range of the Co-doped-g-C_3_N_4_ colorimeter indicate high sensitivity and good accuracy and precision in detecting and measuring the concentration of Hx in aquatic products. Mustafa and Andreescu [132] synthesized a robust, reagentless colorimetric device for monitoring the freshness of meat and predicting spoilage by measuring the level of hypoxanthine. The study entrapped xanthine oxidase and nitro blue tetrazolium chloride (NBT) in a sol–gel biohybrid, resulting in a low LOD of 3.7 μM for hypoxanthine. The low LOD of the reagentless colorimetric device indicates high sensitivity in detecting hypoxanthine in meat samples. Mustafa et al. [133] fabricated an enzyme mimetic nanocatalyst with multiple functions as a peroxidase mimic, a chromogenic indicator, and a redox amplifier, cerium nanoparticles (CeNPs), and a xanthine oxidase (XOD)-based biosensor for monitoring and measuring hypoxanthine. CeNPs and XOD were immobilized on silanized paper and showed a LOD of 15 μM with a linear range up to 800 μM. The low LOD and wide linear range of the CeNPs and XOD colorimeter indicate a high sensitivity and good accuracy and precision in detecting and measuring the concentration of hypoxanthine.

Guo et al. [52] also demonstrated a colorimetric method for the detection of hypoxanthine (Hx) in sea bass fish based on the peroxidase activity of xanthine oxidase grade I ammonium sulfate suspension (XOD-ASS). This method has good selectivity, is low-cost, and is easy to prepare. The LOD was shown to be 6.93 μM, and the technique displayed a good linear relationship in the range from 20 to 200 μM. The color changes from colorless to blue with the presence of H_2_O_2_. The low LOD and wide linear range of the XOD-ASS colorimeter indicate high sensitivity and good accuracy and precision in detecting and measuring the concentration of Hx in sea bass. Ding et al. [134] developed portable silver-doped Prussian blue nanoparticle hydrogels (SPB NPs) incorporated with agarose hydrogels for trimethylamine (TMA) detection in shrimp and fish. The linear range was shown to be from 0.21 to 0.54 ppm. The use of a smartphone and hand-held thermal imager significantly enhances the mobility and usability of on-site monitoring. The narrow linear range of the SPB NPs colorimeter indicates good accuracy and precision in detecting and measuring the concentration of TMA in shrimp and fish. In terms of analytical performance, all five colorimeters described previously have good sensitivity, accuracy, and precision in detecting and measuring the concentration of different substances in various types of samples. The Co-doped-g-C_3_N_4_ reagentless, CeNPs, XOD, XOD-ASS, and SPB NPs colorimeters have low LODs and wide linear ranges, indicating high sensitivity, good accuracy, and precision in detecting and measuring the concentrations of Hx and TMA, respectively. The reagentless colorimeter has a low LOD and no specified linear range, indicating high sensitivity in detecting hypoxanthine in meat samples. Overall, these colorimeters provide reliable and efficient methods for detecting and measuring the concentration of various substances in different types of samples, making them useful tools in various applications, such as food safety, biomedical diagnostics, and environmental monitoring.

**Table 3:** Recent trends of optical biosensors in detecting meat freshness and their analytical performance in food applications.

Sensor	Detection Method	Nanomaterials	Sample	Analyte	Linear Range	LOD	References
Fluorescent-TPE- HPro/XO	Colorimetric	-	Fish	Hx	5–120 μM	1.2 μM	[10]
O-CDs	Colorimetric	Carbon dots	Fish	Hx	2–250 µM	0.61 µM	[24]
XOD-ASS	Fluorescence	-	Fish	Hx	20–100 μM	6.93 μM	[52]
Fluorescent-PtNPs	Fluorescence	Platinum nanoparticles	Fish, shrimp, squid	Hx	8–2500 μM	2.88 μM	[121]
Fluorescent-NH_2_-Cu-MOF nanosheet	Fluorescence	Metal organic frameworks (MOF) nanosheet	Fish	Hx	10–2000 μM	3.93 μM	[123]
DNA-F/GO	Fluorescence	Graphene oxide (GO)	Beef	ATP	20–3500 μM	3.2 μM	[124]
Cys-CuNCs	Fluorescence	Copper nanoclusters	Fish	Hx	8–400 μmol/L	0.7 μmol/L	[125]
XOD@ZnO nanomaterials	Colorimetric	Zinc oxide (ZnO) nanomaterials	Fish	Xa	2.67 × 10^−6^–2.67 × 10^−4^ mol L^−1^	1.30 × 10^−10^ mol L^−1^	[126]
Silver ions and β-D-GP	Fiber-optic	-	Fish	BAs	28.5–114.9 × 10^−9^ mol dm^−3^	2.77 × 10^−9^ mol dm^−3^	[127]
AgP	Colorimetric	Silver nanoplates (AgP)	Fish	Xa	0.15–0.60 μM	0.011 μM	[129]
Paper-based colorimetric biosensor	Fluorescence	-	Pork, chicken, fish meat and fish sauce	Hx	5–40 mg/L	1.8 mg/L	[130]
Co-doped-g-C_3_N_4_	Colorimetric	-	Fish	Hx	2.50–153.1 mg/kg	1.84 mg/kg	[131]
XO/NBT/sol–gel biohybrid	Colorimetric	-	Tilapia fish	Hx	-	3.7 μM	[132]
CeNPs/XOD/silanized paper	Colorimetric	Cerium oxide nanoparticles (CeNPs)	Degraded fish	Hx	800 μM	15 μM	[133]
SPB NPs/Agarose hydrogel	Fluorescence/ colorimetric	Silver-doped prussian blue nanoparticles (SPB NPs)	Shrimp, fish	TMA	0.21–0.54 ppm	-	[134]
Colorimetric-CTAB- Au nanorods	Fluorescence	Gold nanorods (GNrs)	Fish	Hx	0–1130 μM	-	[135]

## 5. Comparison between Conventional Techniques and Nanotechnology Biosensor

Several conventional and biosensor techniques have been discussed in the earlier part. Both techniques can be used to monitor, detect, and evaluate food samples in the food industry. The use of conventional techniques is indeed an indisputable method due to its high selectivity and sensitivity, as discussed above. However, there are some limitations in guaranteeing the on-field and real-time monitoring of the food samples in order to maintain the safety and quality of the food. For instance, capillary electrophoresis has limited utility due to its high cost and low reproducibility; only a select few companies manufacture capillary electrophoresis equipment [136,137]. High-performance liquid chromatography (HPLC) is a time-consuming and laborious method that can separate individual components from complex mixtures, but it has not been successful in retaining these neutral or ionic polar compounds, and it requires highly trained personnel and extensive sample pretreatment [138]. The absorption spectra for Hx, X, and UA all showed significant overlap, making the simultaneous determination of all three components in a biological sample a challenging task [139]. Numerous methods have been reported to enable low-cost, user-friendly, rapid, dependable, selective, and sensitive hypoxanthine detection, which would help address the aforementioned issues.

Nanotechnology biosensors are reported to have few incontestable advantages over conventional techniques, especially with the fabrication of nanomaterials that enhanced the electrochemically active surface area and the efficiency of electron transfer in biosensor sensing systems [140,141], making them particularly useful for the immobilization of enzymes. Additionally, the application of nanotechnology to the analytical performance of electrochemical sensors has vastly improved in terms of low detection limits, broad linear ranges, and high sensitivities. These are the several advantages of nanobiosensors: (1) good selectivity with direct detection of samples or analytes without sample pretreatments or shorten the sample pretreatment process, (2) fast response time within a few seconds to few minutes, (3) low cost as compared to conventional techniques, which required huge cost of instruments for maintenance, (4) perspectives for miniaturization, (5) allow portability, (6) satisfactory analytical performances parameters, and (7) does not required trained personnel to handle equipment or instruments.

In comparing the analytical performance of conventional techniques and nanotechnology-based biosensors in detecting meat freshness (Table 4), it is evident that both types of techniques have good sensitivity and accuracy in detecting various analytes that are indicative of meat freshness. However, there are some differences in the analytical performance of these two types of techniques. One key difference is the linear range of detection. In general, nanotechnology-based biosensors tend to have wider linear ranges of detection compared to conventional techniques. For example, the linear range of detection for xanthine in fish meat using amperometry (XODNPs/Au) is 0.01–1.0 μM, while the linear range of detection using HPLC-UV is 0.05–300 mg/L. Similarly, the linear range of detection for hypoxanthine in fish using fluorescence-NH_2_-Cu-MOF nanosheet is 10–2000 μM, while the linear range of detection using HPLC-UV is 0.05–300 mg/L. This wider linear range of detection may make nanotechnology-based biosensors more suitable for detecting a wider range of analyte concentrations. Another difference is the limit of detection (LOD). In general, nanotechnology-based biosensors tend to have lower LODs compared to conventional techniques. For example, the LOD for xanthine in fish meat using amperometry (XODNPs/Au) is 0.01 μM, while the LOD using HPLC-UV is 0.0774 mg/L. Similarly, the LOD for hypoxanthine in fish using fluorescence-NH_2_-Cu-MOF nanosheets is 3.93 μM, while the LOD using HPLC-UV is 0.0555 mg/L (based on Table 4). This lower LOD may make nanotechnology-based biosensors more suitable for detecting trace levels of analytes.

In conclusion, both conventional techniques and nanotechnology-based biosensors have good sensitivity and accuracy in detecting various analytes in meat samples, but there are some differences in their analytical performance. Nanotechnology-based biosensors tend to have wider linear ranges of detection and lower limits of detection compared to conventional techniques. This may make them more suitable for detecting a wider range of analyte concentrations and trace levels of analytes, respectively. However, it is important to note that the choice of technique ultimately depends on the specific requirements and constraints of the application, and both types of techniques have their own advantages and limitations.

## 6. Conclusion and Future Perspectives

In conclusion, the use of nanotechnology-based biosensors for the assessment of meat freshness offers several advantages over traditional methods. These sensors are highly sensitive and specific and are able to detect very low concentrations of contaminants and distinguish between different types of purine derivatives. They also have a rapid response time, providing results in a matter of minutes or even seconds, which allows for more efficient and effective monitoring of meat products. Additionally, nanotechnology-based biosensors are portable and easy to use, making them well-suited for on-site testing in food processing plants or other settings.

However, while the use of these sensors has the potential to revolutionize the way we assess meat freshness, it is important to consider the potential challenges and limitations. One potential concern is the cost of purchasing and maintaining these sensors, which may be more expensive than traditional techniques. It is also important to consider the potential impact on the workforce, as the widespread adoption of these sensors could potentially lead to job losses in certain sectors. Overall, it is clear that the world is well-prepared for the lightning-fast pace of nanotechnology-based biosensors for the assessment of meat freshness, but it is important to approach their adoption with caution and consideration of all stakeholders.

## Figures and Tables

**Table 1:** Conventional methods for detecting purine and their analytical performance in food applications.

Analysis Method	Sample	Analyte	Linear Range	LOD	Reference
HPLC-DAD	Cow milk	Allantoin, Uric acid, Xa, Hx	3.125–100 µg/mL	0.74 µg/mL 0.16 µg/mL 0.09 µg/mL 0.14 µg/mL	[16]
HPLC-UV	Fish, shellfish, clam	Adenine, Guanine, Hx, Xa	0.05–300 mg/L	0.02 mg/L 0.03 mg/L 0.06 mg/L 0.10 mg/L	[45]
CZE	Salted herring meat	Trp, Thr, Met, Phe, Tyr	-	-	[46]
UHPLC-MS	Chicken	Histidine, Hx, Inosine	-	-	[47]
UHPLC-MS	Serum	Twenty-three purine derivatives	0.002–11.2 μg/mL	0.05–6.3 ng/mL	[48]
GCMS	Beef	Glutamine, Adenosine, Hx	-	-	[49]
UPLC-MS	Fresh tuna fish	Uracil, Inosine, Hx	-	-	[50]
Spectrophotometric	Cattle meat	Adenosine, Inosine, Hx	-	-	[51]
HPLC-UV	Umami soup stock	Inosine monophosphate, Guanosine monophosphate, Hypoxanthine, Inosine	-	-	[53]
Reverse phase HPLC	Processed chicken meat	Adenine, Guanine, Hx, Xa	-	-	[54]
HPLC-VWD	Chinese chicken broth	Adenine, Guanine, Hx, Xa, Uric acid	0.05–100 mg/L	0.66 µg/L 0.64 µg/L 0.58 µg/L 1.14 µg/L 1.71 µg/L	[55]
HPLC-UV	Shiitake mushroom	Guanine, Adenine, Hx, Xa	-	-	[56]
CZE	Yeast (*Saccharomyces cerevisiae*)	Adenine	1–20 mg/L	1.11 μg/L	[57]
CZE-UV	Beer	Hx, Xa, Adenine, Thymine	0.4–40 mg/L	0.1 mg/L 0.1 mg/L 0.1 mg/L 0.3 mg/L	[58]
CE-UV	Soybean milk	Adenine, Guanine, Xa, Hx	0.5 to 100 mg/mL	0.08 μg/mL 0.06 μg/mL 0.09 μg/mL 0.05 μg/mL	[59]

**Table 4:** Comparison of the analytical performance of conventional techniques and nanotechnology-based biosensors.

Analysis Method	Sample	Analyte	Linear Range	LOD	Reference
**Conventional**					
HPLC-DAD	Cow milk	Allantoin, Uric acid, Xa, Hx	3.125–100 µg/mL	0.74 µg/mL 0.16 µg/mL 0.09 µg/mL 0.14 µg/mL	[16]
Au-PEDOT-fMWCNT/GCE	Fish meat	UA Xa Hx	0.1–800 μM 0.05–175 μM 0.1–150 μM	199.3 nM 24.1 nM 90.5 nM	[20]
HPLC-UV	Marine fish	Adenine, Guanine, Hx, Xa	0.1–300 mg/L	0.0774 mg/L 0.0178 mg/L 0.0118 mg/L 0.0555 mg/L	[40]
HPLC-UV	Raw anchovies	Adenine, Guanine, Hx, Xa	-	-	[44]
HPLC-UV	Fish, shellfish, clam	Adenine, Guanine, Hx, Xa	0.05–300 mg/L	0.02 mg/L 0.03 mg/L 0.06 mg/L 0.10 mg/L	[45]
UHPLC-MS	Serum	23 of purine derivatives	0.002–11.2 μg/mL	0.05–6.3 ng/mL	[48]
HPLC-VWD	Chinese chicken broth	Adenine, Guanine, Hx, Xa, Uric acid	0.05–100 mg/L	0.66 µg/L 0.64 µg/L 0.58 µg/L 1.14 µg/L 1.71 µg/L	[59]
**Nanotechnology based biosensor**					
Amperometry (XODNPs/Au)	Fish meat	Xa	0.01–1.0 μM	0.01 μM	[84]
XO/Poly(l-Asp)/MWCNT/GCE electrode	Fish meat	Xa	0.001–0.004 μM	3.5 × 10^−4^ μM	[96]
Fluorescent-NH_2_-Cu-MOF nanosheet	Fish	Hx	10–2000 μM	3.93 μM	[123]
CeNPs/XOD/silanized paper	Degraded fish	Hx	800 μM	15 μM	[133]

## Data Availability

Not applicable.
